# Indicators for Universal Health Coverage: can Kenya comply with the proposed post-2015 monitoring recommendations?

**DOI:** 10.1186/s12939-014-0123-1

**Published:** 2014-12-20

**Authors:** Valerie Obare, Claire E Brolan, Peter S Hill

**Affiliations:** School of Population Health, The University of Queensland, Public Health Building, Herston Rd, Herston, Brisbane, QLD 4006 Australia

**Keywords:** Universal health coverage, WHO/World Bank framework, Monitoring, Indicators, MDGs, Chronic conditions and injuries, Kenya

## Abstract

**Introduction:**

Universal Health Coverage (UHC), referring to access to healthcare without financial burden, has received renewed attention in global health spheres. UHC is a potential goal in the post-2015 development agenda. Monitoring of progress towards achieving UHC is thus critical at both country and global level, and a monitoring framework for UHC was proposed by a joint WHO/World Bank discussion paper in December 2013. The aim of this study was to determine the feasibility of the framework proposed by WHO/World Bank for global UHC monitoring framework in Kenya.

**Methods:**

The study utilised three documents—the joint WHO/World Bank UHC monitoring framework and its update, and the Bellagio meeting report sponsored by WHO and the Rockefeller Foundation—to conduct the research. These documents informed the list of potential indicators that were used to determine the feasibility of the framework. A purposive literature search was undertaken to identify key government policy documents and relevant scholarly articles. A desk review of the literature was undertaken to answer the research objectives of this study.

**Results:**

Kenya has yet to establish an official policy on UHC that provides a clear mandate on the goals, targets and monitoring and evaluation of performance. However, a significant majority of Kenyans continue to have limited access to health services as well as limited financial risk protection. The country has the capacity to reasonably report on five out of the seven proposed UHC indicators. However, there was very limited capacity to report on the two service coverage indicators for the chronic condition and injuries (CCIs) interventions. Out of the potential tracer indicators (n = 27) for aggregate CCI-related measures, four tracer indicators were available. Moreover the country experiences some wider challenges that may impact on the implementation and feasibility of the WHO/World Bank framework.

**Conclusion:**

The proposed global framework for monitoring UHC will only be feasible in Kenya if systemic challenges are addressed. While the infrastructure for reporting the MDG related indicators is in place, Kenya will require continued international investment to extend its capacity to meet the data requirements of the proposed UHC monitoring framework, particularly for the CCI-related indicators.

**Electronic supplementary material:**

The online version of this article (doi:10.1186/s12939-014-0123-1) contains supplementary material, which is available to authorized users.

## Introduction

### Background on Universal Health Coverage

Universal Health Coverage (UHC) has been defined as providing access to needed health services without incurring financial hardships for the whole population [1], and is receiving renewed attention at both global and national levels. In 2005 the Member States of the World Health Organization (WHO) adopted a resolution encouraging countries to develop health financing systems aimed at achieving UHC [2]. Recently, the quest for countries to achieve UHC has received significant support from key global players, with the WHO, World Bank and United Nations General Assembly all making commitments to the UHC agenda [3,4]. The fact that millions of people still lack access to basic health care services motivates this attention [5]. Similarly, the costs associated with utilising health services place an immense financial burden on many households. Global estimates indicate that every year, nearly 150 million people experience catastrophic health expenditure where household out-of-pocket payments for health care consume such a proportion of their income that it forces them to forego other goods and services [6], while 100 million are pushed into poverty [1].

UHC is increasingly embraced at a global level as a priority in the post-2015 development agenda [3,7]. Health is acknowledged as essential for human welfare and sustained economic and social development [1]. When people have poor health, with lack of health service being a one of the contributing factors, they often are vulnerable to poverty. At the same time, people seeking health services may incur impoverishing health costs [1]. This paradox provides an affirmation of the critical link between health, sustainable development and economic growth [8]. Ill health affects productivity and diverts households’ income to seeking health services, thus negatively impacting on economic and social development [5,9]. Achieving UHC is primarily an issue of equity, ensuring that people can access the health services they need to keep them healthy and productive, while at the same time, safeguarding them from being pushed into poverty due to out-of-pocket health expenditures [3]. UHC strategy will contribute to improving health as well as reducing the vulnerability to poverty; thus contributing to the post-2015 agenda on sustainable development.

To progress towards UHC, countries will need to concurrently undertake health financing reforms as well as comprehensively address health systems service delivery challenges [8,10]. According to the WHO 2010 report, the UHC target is to progressively expand the range of health services offered, the proportion of the population covered and the proportion of health cost covered to reduce the financial burden on households [1]. The WHO 2010 report identified three critical areas for health financing reforms. These reforms require raising necessary health funds to offer health services, shifting to viable pre-payment methods and improving efficient and equitable use of available health resources [1]. The strategies that countries adopt to achieve UHC vary [11]. Country-specific contexts i.e. disease burden, health system, economic as well as political factors, will greatly influence the policy choices, but in spite of the varied approaches to achieving UHC, the three dimensions of UHC will apply across all contexts[12]. These cross-cutting aspirations of UHC form the foundation for measuring progress.

For WHO, monitoring progress towards UHC is one of its research priorities, and will facilitate assessment and tracking of strategies implemented and their outcomes. A global monitoring framework will allow joint learning and sharing of experience and knowledge on UHC implementation across different contexts, and a common and comparable approach in assessing UHC progress is currently being developed [11,13].

In recent years, consultative meetings have been conducted to develop a common mechanism of monitoring progress towards UHC. These meetings addressed the concepts of UHC that will be measured, and described the potential indicators to be utilised [14-16]. There is a consensus that measurement of UHC will primarily focus on the level and distribution of the service coverage and financial protection as well as ensuring equity [16]. The culmination of these discussions resulted in the release of the joint WHO/World Bank paper in December 2013, proposing a framework for tracking UHC progress at a national and global level. The aim of the framework is to foster a common approach to measuring country progress against standardised international indicators. This will facilitate comparison of the progress made towards UHC among different countries so that they can learn from each other.

Recent studies on the measurement of UHC progress have explored possible indicators for UHC, the availability of the indicators in low income countries, and validity of commonly proposed indicators and the data sources [17-19]. The approaches of these studies, however, were not based on the joint WHO/World Bank framework. In order to inform the continuing development of that framework, this study seeks to assess Kenya’s ability to report on the WHO/World Bank UHC indicators. The paper describes the current context of UHC in Kenya; identifies the available tracer indicators for the proposed framework; identifies the data sources for the indicators; and describes the factors that will affect the feasibility of the WHO/World Bank framework in that country. The findings from this study will contribute to the on-going discussions on measuring progress toward UHC by highlighting the factors that will affect implementation and applicability of the proposed framework in Kenya.

### Background on the demographic and socio-economic status of Kenya

Kenya is a low income country, with slow economic growth. The main economic sectors include agriculture, tourism and service industry [20]. The population has increased four-fold since Independence in 1963 to about 43 million people (2012). This rapid population growth has placed an enormous strain on the limited resources for health services [21]. The majority of Kenyans live in rural areas and mainly depend on subsistence farming for their livelihood [20]. Nearly half of the population live in poverty and are vulnerable to poor health [20]. Table 1 illustrates a summary of key demographic and socio-economic indicators in the country. Kenya, like other low income countries, has limited national resources, a significant proportion of the population living below the poverty line and a high disease burden; highlighting the complex landscape in which UHC will be implemented. The fact that the majority of Kenyans are poor and vulnerable suggests the country’s government will need to adopt policies that reflect and respond to this reality. Certainly, most poor households in Kenya are unable to make any form of payment for health services without incurring a financial burden [1,5].

**Table 1:** **Summary of demographic and socio-economic indicators for Kenya**

**Indicator**	**Value**
Total population (2012)	43.18 million
Urban population (2011)	24%
Life expectancy at birth (years) (2012)	61
Total fertility rates (births per woman) (2014)	3.54
GNI per capita (2012)	US$ 860
GDP growth (2012)	4.6%
Unemployment rates (2008)	40%
Population living below the poverty line (2012)	43.4%

Sourced from World Bank and Centre for Intelligence Agency (CIA) [20,22].

Year in brackets indicates most recent data available.

### Overview of Kenya’s Health System

#### Disease burden

The country grapples with a high disease burden, of which the traditional communicable diseases are the major cause [23]. The Global Burden of Disease Study (2010), for example, indicates that communicable diseases, maternal, neonatal and nutritional conditions remain the top ten leading causes of Disability Adjusted Life Years (DALYs) in Kenya [24]. However, non-communicable diseases (NCDs) and injuries are increasingly becoming an important contributor to the disease burden [23]. Further analysis suggests that, apart from HIV/AIDS, NCDs and injuries represent the leading cause of DALYs among adults [25]. As the country makes gains in the control of HIV/AIDS, the significant burden of NCDs and injuries among adults is thus a growing concern among health policy makers [26].

#### Service delivery and health outcomes

In Kenya health services are provided by four main sectors: public, private, faith-based and non-governmental organisations (NGOs). The private and faith-based institutions are a mix of profit and not-for-profit agencies. The public sector operates the largest share of healthcare facilities in the country, and is the major health service provider in the rural areas [27,28]. As such, access to health services by the majority of Kenyans is largely influenced by the functionality of the public health sector.

WHO has described six health systems functions i.e. health financing, service delivery, health workforce, information, medical products, vaccines and technologies, leadership and governance, defining the desirable attributes of a health system to facilitate adequate service coverage [29]. The Kenyan public health sector faces numerous challenges in service delivery that affects all six health system functions [21,30]. The sector is characterised by inadequate and mismanaged funding, inefficiencies, shortage of health workers, inadequately equipped facilities, medicine stock outs; hence limiting the availability and quality of health services [27,30]. Table 2 presents a summary of key service delivery, health financing and health outcome indicators. The performance of the country’s health sector indicators is generally comparable to other low income countries, and issues being addressed by Kenya in working towards UHC are shared by other low income countries. The current per capita total health expenditure (US$ 42.2) is still below the estimated cost of implementing UHC (US$ 60 per capita) [1,31]. The limited availability of funds has adversely impacted on all the other functions of the health system resulting in limited access to and quality of health services and inefficient utilisation of available resources by Kenya’s Ministry of Health has further exacerbated inequitable service delivery [30].

**Table 2:** **Summary of key Kenyan health sector indicators values in comparison to average values for low income countries**

	**Indicators**	**National value**	**Average for low income countries**	**Source**
Health Service delivery	Births attended by skilled health personnel (%) (2005–2012)	44	47	WHO
	Contraceptive Prevalence (%) (2005–2012)	46	38	
	Neonates protected at birth against neonatal tetanus (%)(2011)	73	82	
	DPT3 Immunisation coverage among 1-year-olds (%)(2011)	88	79	
	Density of nursing and midwifery personnel per 10 000 population	7.9	14.9	
	Hospitals (per 10 000 population)	1.6	0.9	
	Median availability of selected generic medicines in public sectors (%)	37.7%	No data	
Health financing	Total expenditure on health as a percentage of gross domestic product (2010)	5.4%	5.3%	Kenya NHA and WHO
	Per capita expenditure on health expenditure on health at average exchange rate (US$)	42.2	28	
	General government expenditure on health as a percentage of total expenditure on health	28.8%	38.5%	
	Government expenditure on health as a percentage of total government expenditure	4.6%	9.3%	
	Private expenditure on health as a percentage of total expenditure on health	37%	61.5%	
	Out-of-pocket expenditure as % of private expenditure on Health	76.6%	77.7%	
Health status	Maternal mortality ratio (per 100 000 live births) (2010)	360	410	WHO
	Under five mortality rate (per 1,000 live births) (2011)	73	63	
	Adult mortality rate (probability of dying between 15–60 years of age per 1000 population) (2011)	Male = 346	Male = 288	
		Female = 294	Female = 245	

Sourced from WHO and Kenya National Health Accounts (NHA) [31,32].

## Methods

### WHO/World Bank UHC monitoring framework

**Figure 1:**
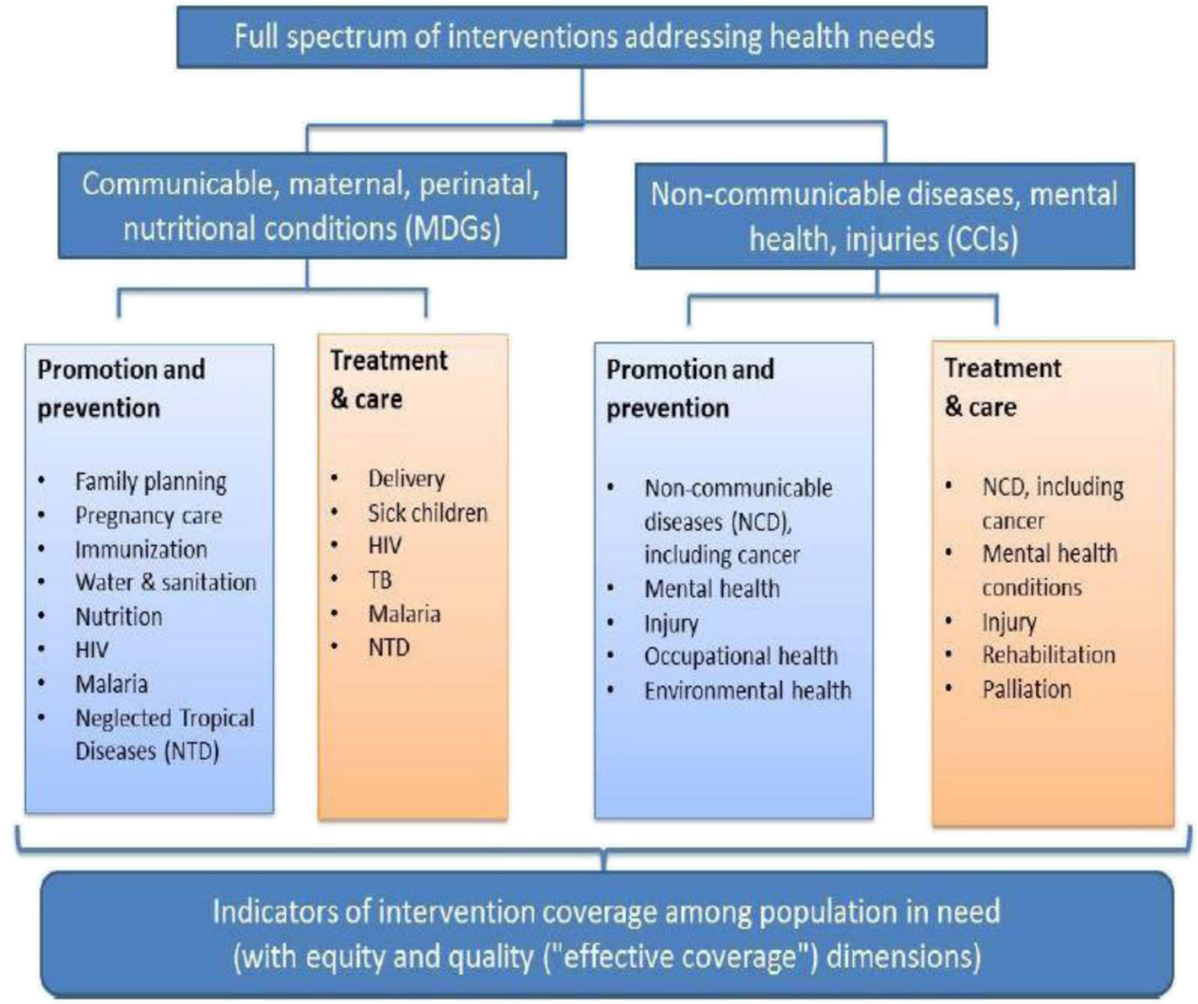
**Framework for selecting indicators to monitor service coverage.** Source WHO/World Bank [12].

This study utilises three documents to benchmark Kenya’s capacity to report on UHC monitoring indicators: the proposed WHO/World Bank UHC monitoring framework [12], the subsequent meeting report sponsored by the WHO and Rockefeller Foundation held in Bellagio in 2012 [16], and the WHO *Draft for comprehensive global monitoring framework and targets for the prevention and control of noncommunicable diseases (NCDs)*, which further developed indicators for NCDs [33]. Together, these documents provided the list of potential indicators that were used to determine the feasibility of the proposed framework. Performance indicators were identified in each document; duplicates removed, and where the WHO/World Bank committee developed preferred variants (e.g. “households protected” from, rather than “households incurring” impoverishing and catastrophic expenditure), their preferred new variant was accepted. The WHO/World Bank UHC monitoring framework focuses on two discrete treatment components for the measurement of UHC progress (see Table 3): first, the level of service coverage; and second, financial risk protection. Health service coverage indicators are separated into two measurement components, and clearly distinguish between the current MDG-related interventions and proposed indicators for chronic conditions and injuries (CCIs) related interventions. The health service coverage measures also include a broad set of intervention indicators that capture prevention and promotion services as well as services across the different levels of the health system. Figure 1 graphically represents the framework for selecting indicators to monitor service coverage [11,15]. The list of potential tracer indicators aggregated from our three sources is outlined in Additional file 1 and Additional file 2, [12].

**Table 3:** **Global-level framework for monitoring UHC**

Goal	Achieve UHC – All people should have access to the quality, essential health services they need without enduring financial hardship
Target	By 2030, at least 80% of the poorest 40% of the population have coverage to ensure access to essential health services
	By 2030, everyone (100%) has coverage to protect them from financial risk, so that no one is pushed into poverty or kept in poverty because of expenditure on health services
Health service coverage indicators	MDG-related indicators:
	1. Aggregate: A measure of MDG-related service coverage that is an aggregate of
	2. single intervention coverage measures
	3. Equity: A measure of MDG-related service coverage for the poorest 40% of the population
	CCIs-related indicators:
	1. Aggregate: A measure of CCIs-related service coverage that is an aggregate of single priority interventions to address the burden of NCDs, including mental health and injuries
	2. Equity: A measure of CCI service coverage for the poorest 40% of the population
Financial risk protection indicators	Impoverishing Expenditure:
	1. Aggregate: A measure of the level of household impoverishment arising from out-of-pocket expenditures on health, equal to the ratio of the poverty gap in a world without out-of-pocket payments to the actual (larger) poverty gap.
	Catastrophic Expenditure:
	2. Aggregate: The fraction of households incurring catastrophic out-of-pocket health expenditures.
	3. Equity: The fraction of households among the poorest 40% of the population incurring catastrophic out-of-pocket health expenditures.

Sourced from WHO/World Bank [12].

### Literature search strategy

A comprehensive literature search was undertaken to identify literature describing UHC in Kenya and to identify potential sources for monitoring UHC. The search was done in two phases. The first phase captured literature that addressed the two UHC components (service coverage and financial protection) in Kenya. The second phase captured literature that addressed the measurement of UHC indicators. Table 4 illustrates the different search terms used. The literature search was conducted between February to April 2014 utilising the Pub Med, CINAHL, Scopus, Web of Science and Google scholar databases. A further web-based search was conducted for relevant grey literature i.e. government policy documents, sessional papers, legislative bills and reports. The websites accessed include WHO, World Bank, Kenyan Government websites, UHCforward.org, Institute of Health Metrics and Evaluation (IHME), Measure DHS, Health systems 20/20, the Joint Learning Network for Universal Health Coverage and Universal Health 2030. In addition, the reference lists of the key articles and reports were further scrutinised to identify more articles.

**Table 4:** **Summary of the search terms used**

	**Search terms**	**Geographical**
First phase	“health sector reforms”, “universal health coverage”, “health financing” “universal access to health care” “health insurance”, “out-of- pocket expenditure”, “health care cost” “Health system reforms”	“Kenya”, “developing countr*”, “low income countr*”,
Second phase	“health information system” “health metrics” “monitor* universal health coverage” “health indicator*” “measur* universal health coverage” “MDG monitor*” “NCD indicator*”	

*in search terms indicates all variants of the term, eg measur* will identify measure, measures, measurement, measurements; countr* will identify country, countries.

#### Selection of articles

The search was conducted purposively to identify key relevant resources to answer the research objectives. The abstract and summaries of the identified articles and reports published in English were reviewed to determine the relevance of the documents in relation to the research question. A total of 228 documents were retrieved into Endnote referencing software. The retrieved documents were then reviewed to identify and exclude duplicates, and then to select the sources providing information on relevant potential data sources, using the following inclusion criteria:

- Published in the year 2000 onwards
- Published in English Language
- Research studies conducted in Kenya
- Literature addressed health service coverage, financial risk protection, measurement of UHC and health information systems
- Kenyan Government policy documents and reports that addressed access to health services and health information systems

**Figure 2:**
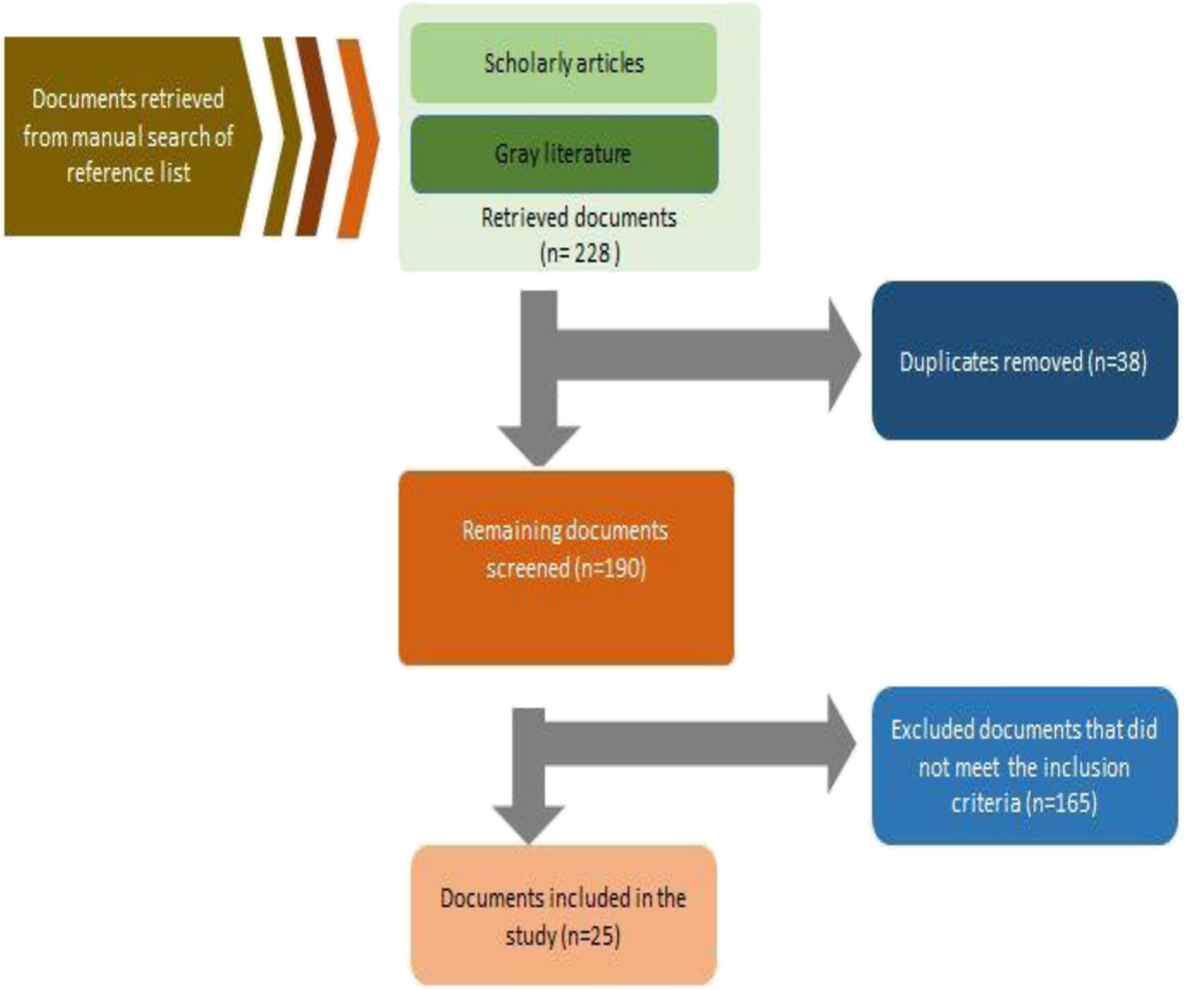
**Flowchart demonstrating the processes for reviewing literature.**

A total of 25 documents were included in the study, listed in Additional file 3. These provided information on the availability of data for tracer indicators and its frequency of collection, but did not provide an assessment of the quality of the data. Figure 2 presents a flowchart to illustrate the process of reviewing the literature.

### New developments on the WHO/World Bank UHC monitoring framework

Since commencing the study, the WHO/World Bank framework was recently revised to capture the feedback received from various stakeholders [33]. The principles and the concepts to be monitored i.e. level and distribution of service coverage; financial protection and equity remain the same. The revised framework adopted limited changes in terms of the indicators to be used, and these have been incorporated into our analysis. However the presentation of the indicators has been reconfigured. Firstly, the health services coverage indicators, initially presented as aggregate MDG-related and CCI-related measures, have been integrated to represent aggregate prevention and treatment measures. Secondly, equity measures are to be disaggregated by place of residence, gender, and wealth quintile across the whole population. Lastly, the financial protection coverage indicators have been refined to measure the “households protected” from, rather than “households incurring” impoverishing and catastrophic expenditure due to out-of-pocket health expenditures [12,34]. Additional file 4 summarises the revised WHO/World Bank framework for monitoring UHC.

This study utilised both versions of the joint WHO/World Bank framework [12,34], together with the report from the Bellagio meeting [16]. We have retained the structure from the first version of the framework to distinguish between data sources and gaps that have been established for the current MDG-related measures and those required for proposed CCI-related measures. The revised framework was utilised for the analysis of the new dimensions of coverage, disaggregation of equity measures, and the shift to reporting on “households protected”.

## Findings and discussion

### UHC in the Kenyan context

**Figure 3:**
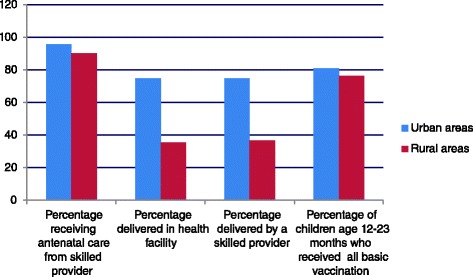
**Differentials between urban and rural populations for key MCH services.** Sourced from KDHS [39].

Kenya is in the initial stages of implementing UHC [11,35]. The existence of policy strategies and a rudimentary National Health Insurance Fund (NHIF) suggest that some attention has been made to address the improved access and financial protection aspects of UHC [26,35]. However, there are still major weaknesses in the health system that results in skewed access, disproportionately disadvantaging the rural and poorer populations. Figure 3 illustrates the differences in some of the health indicator between urban and rural populations [36-38]. Current progress against the three aspects of UHC in Kenya has been summarized from the literature, in particular a nationally representative cross-sectional household survey [28] and presented in Table 5. Currently, a significant majority of the population do not have access to needed health services. The Kenyan health sector is significantly dependent on out of pocket payments for health services [31], and health care costs are increasingly impoverishing Kenyan households and pushing some households into poverty [36]. Such results indicate the country needs to urgently invest in and implement policies that will facilitate progress towards UHC aspirations.

**Table 5:** **Summary of UHC aspects in the Kenyan context**

**UHC aspects**	**Description**
Access to needed services	• The right to health services by all Kenyans is articulated in the constitution
	• A standard Kenya essential health package is being implemented and has included interventions for non-communicable diseases and injuries as well.
	• The availability of health facilities and services are limited, more so for the rural population.
	• The range and quality of health services offered are limited.
	• Cost is a key barrier to accessing health services.
Financial risk protection	• Existing health financing mechanisms offer very limited financial risk protection.
	• Out of pocket expenditure is major source of health sector financing in the country.
	• Nearly 10% of Kenyans have access to any form of health insurance. Majority of the health insurance schemes require co-payments for medicine or certain outpatient and diagnostic services thus offering limited protection.
	• Kenyan households incur impoverishing and catastrophic health expenditure. Estimates suggest that nearly 1.5 million households are pushed below the national poverty line due to health care payments.
Equity	• Health sector is inequitable. The distribution and utilisation of health services favour the wealthier and urban populations in the country.

Sourced from [23,26-28,30,31,38,40-42].

#### Policy framework for UHC in Kenya

The country has yet to establish a formal policy declaration on UHC that is entrenched in legislation. The current *Kenya Health Policy 2012–2030* is the most detailed policy document that addresses certain aspects of UHC [23]. The policy objective “*to attain universal coverage of critical services that positively contribute to the realisation of policy goals*” provides a documented commitment to achievement of UHC for all Kenyans [23]. Similarly, the country has undertaken various strategies to facilitate improved access to affordable health services and address the high disease burden [26,43]. The most significant strategies in relation to UHC are the two attempts to transform the country’s NHIF into a compulsory social health insurance [21,44]. The objective of these proposed amendments was to shift the current health financing arrangements to prepayment mechanisms, reducing the dependence on out of pocket payment and mobilising more funds into the health sector through membership contributions. The proposed amendments were not adopted into legislation due to the lack of finances to cater for the cost of the scheme, and opposition from a section of professional bodies, employers and the private health sector [21,44]. Although the proposed amendments were not adopted into legislation, implementation of social health insurance remains a priority agenda in the Ministry of Health’s strategic plan [26]. Discussions are still ongoing to determine a viable approach of ensuring that Kenyans have access to social health insurance coverage.

### Feasibility of the proposed UHC monitoring framework in Kenya

#### MDG-related service coverage indicators

From critical examination of the literature, Kenya has the capacity to report on the majority of the proposed indicators for MDG related service coverage. The capacity to report implies that the country has established a network for reporting the MDG-related in the public sector, some health workers are trained on the reporting process. However, several challenges exist in relation to quality, timeliness of the data generated currently exist [30,45]. Key data sources for MDG related indicators are the Kenya AIDS Indicator survey (KAIS), Kenya demographic health survey (KDHS), Kenya service provision assessment survey (KSPA), Kenya Malaria indicator survey and routine facility data. Table 6 illustrates the available MDG related indicators and their respective data sources, though the quality of these indicators cannot be assessed in this study. Out of the potential 22 indicators being considered for global monitoring, only 1 indicator was not available in-country: “Measles, BCG, polio, hepatitis B and Influenza coverage among older people”, and will require establishing screening for older people. In the event that the global-level UHC monitoring prioritises this indicator, Kenya will need to identify a mechanism for collecting the data; possibly consider integrating the indicator into one of the health population surveys conducted in the country.

**Table 6:** **Availability of MDG related service coverage indicators and their data sources**

**UHC Health service coverage indicators indicator**	**Potential tracer indicators for aggregate MDGs-related interventions (n = 22 indicators)**	**Indicator available**	**Data source.**
Aggregate: A measure of MDG-related service coverage that is an aggregate of single intervention coverage measures	Need satisfied for family planning	Yes	Routine facility data/KDHS.
	Skilled birth attendance	Yes	
	DPT3 immunisation coverage	Yes	
	Serious acute child illness coverage (Percentage of Health Facilities providing treatment as per the IMCI guidelines)	Yes	Supervision reports/Kenya service provision assessment survey
	Household ownership of insecticide treated nets (ITNs)	Yes	Malaria Indicator survey
	Tuberculosis treatment coverage	Yes	TB programme reports
	Ante-retroviral treatment (ART) coverage	Yes	routine facility data (NASCOP reports)
	PMTCT service coverage	Yes	
	Additional coverage indicators		
	Contraceptive use	Yes	routine facility data/KDHS
	ANC 4+ visits	Yes	
	Institutional deliveries	Yes	
	Postnatal care visit within two days of childbirth (%)	Yes	
	Measles, BCG, polio, hepatitis B, Influenza coverage among older people	No	
	Suspected pneumonia treated with antibiotic	Yes	routine facility data/KDHS
	Diarrhoea treated with oral rehydration salts (ORS)	Yes	
	Coverage of exclusive breast feeding	Yes	KDHS
	Intermittent prevention treatment (IPT) during pregnancy	Yes	Malaria programme reports/ malaria indicator survey
	Fever treated with antimalarials	Yes	Routine facility data/Malaria indicator survey/KDHS
	Households with indoor residual spraying (IRS).	Yes	Malaria indicator survey
	TB case detection rate (the number of estimated new TB cases detected in a given year using the DOTS approach) expressed as a percentage of all new TB cases)	Yes	TB programme reports
	Male circumcision rates	Yes	KAIS
	Condom use at higher risk sex	Yes	KAIS
Equity: A measure of MDG-related service coverage for the poorest 40% of the population		Yes	Surveys mentioned above.

KAIS: Kenya AIDS Indicator survey; KDHS: Kenya demographic health survey; KSPA: Kenya service provision assessment survey; NASCOP: National AIDS and STI Control Programme.

Data sourced from various Government of Kenya (GoK) documents [22,32,39,43,44,46-50].

The current frequency of generating these indicators varies, depending on the data source and funding. Table 7 indicates the frequency of the various data sources in Kenya. Routine facility data are generated on a monthly basis and provide the most current data. However routine facility data is not representative of the general population, and only captures information from people who attend health facilities. Furthermore, the Ministry of Health HIS reporting channels largely captures data from public health facilities and a few faith based facilities [26]. Routine data from the private health sector is yet to be captured through the national health information system [51]. The major pitfalls of routine facility data are unreliability, inconsistency and incompleteness [30,52], resulting in minimal utilization of the data for policy making [30,43,51]. The surveys apply systematic methods, and data collection is consistent, thus generating more reliable information. While the data is more generalizable to the population since the survey samples are more representative of the population, the surveys generate retrospective data and are often costly to conduct. National surveys in Kenya are currently donor funded and sustained donor funding is critical for these surveys. The health information system in the country is underfunded, and it is unlikely that the Ministry of Health would be unable to sustain these surveys without donor assistance [30,51].

**Table 7:** **Frequency of various data sources in Kenya**

**Survey**	**Last conducted**
Kenya AIDS Indicator survey (KAIS),	
Kenya demographic health survey (KDHS),	2008/2009 follow up of 2003 survey
Kenya service provision assessment survey (KSPA)	2010 survey was a follow up of 2004,1999 survey
Kenya malaria indicator survey	2010 follow up to 2007
Kenya national health accounts survey	2009/10 follow up of 2005/06 survey
Kenya household health expenditure and utilisation survey	2007 follow up to 2003 survey
Census	2009 follow up to 1999 census

Sourced from [27,28,30,31,39,50].

Measurement of the equity indicator for MDG-related service coverage will be possible from the following surveys: Kenya AIDS Indicator survey (KAIS), Kenya demographic health survey (KDHS), Kenya service provision assessment survey KSPA and the census-collect primary data on the socio-economic status of the respondents [30,39]. This will allow for the disaggregation of data on socio-economic status and measuring MDG service coverage among the poorest 40% of the population. However the primary data on socio-economic status are currently only updated every 5–10 years, depending on the periodicity of the surveys.

The revised WHO/World Bank UHC monitoring framework has broadened the dimensions of equity measures to include place of residence and gender in addition to the wealth quintile. But in the country’s current reporting processes, most data from facilities are aggregated at district level. Information available at national level will therefore be aggregate district measures, and further information on place of residence may be more cumbersome to retrieve. Furthermore, due to the frequent mobility of Kenyans in both urban–rural migrants and the pastoralist communities, the place of residence may be difficult to ascertain for the purposes of measuring equity [30].

#### CCI related service coverage indicators

The country has very limited capacity to report on the potential CCI related service coverage indicators. There is a paucity of CCI indicators in the country’s health sector. Table 8 illustrates the available CCI related service coverage indicators and their respective data sources. Only four out of the 27 potential tracer indicators for the aggregate CCI measures are currently available. Moreover, two of the four tracer indicators captured in the policy documents (i.e. “percentage of the population that is overweight and obese” and “percentage of women with cervical cancer screening”) are not currently linked to any reliable data source: a survey of obesity has been proposed, and cervical cancer screening reports are available from a limited number of clinics. The national monitoring framework of the health strategic plan identified the health information system as the source of data for these two indicators [26]. However, a perusal of the currently approved data collecting and reporting tools indicate that such information is not captured in the routine reporting tools. The postnatal (MOH 406) register includes a data entry column for “screened for cervical cancer”. However the main monthly aggregate summary tools i.e. *National Integrated Tool for reproductive health, HIV/AIDS, Malaria, TB and Child Nutrition* (MOH 711B) form and *Monthly Workload Report for Hospitals* (MOH 717 form) do not capture this information, and it is unlikely that the primary data for this indicator is compiled and reported to the national level consistently. To obtain data for the percentage of women screened for cervical cancer would therefore require physical retrieval of data from the postnatal registers [46]. Even with better quality reporting, the data obtained from the postnatal register will only capture the small subset of women who attend the clinics rather than the target population as a whole. As such, the country will be unable to meet the demand of global reporting on the two CCI related service coverage indicators, without significant investments into their reporting.

**Table 8:** **Availability of CCI related service coverage indicators and their data sources**

**UHC Health service coverage indicators**	**Potential tracer indicators for the aggregate CCIs-related service coverage measures (n = 27 indicators)**	**Indicator available**	**Data sources**
Aggregate: A measure of CCIs-related service coverage that is an aggregate of single priority interventions to address the burden of NCDs, including mental health and injuries	Percentage with hypertension diagnosed and receiving treatment	No	
	Probability of dying between the exact ages of 30 and 70 from any of cardiovascular disease cancer diabetes or chronic respiratory disease	No	
	Age-standardised prevalence of diabetes (based on HbA1c levels), hypertension, cardiovascular disease and chronic respiratory disease	No	
	Age-standardised mean population intake of salt (sodium chloride) per day in grams in persons aged 18+	No	
	Prevalence of persons aged 18+) consuming less than five total servings (400g) of fruit and vegetable per day	No	
	Fraction of calories from added saturated fats and sugars	No	
	Hepatitis B vaccination coverage	No	
	Percentage of the population that is overweight and obese	Not Yet	a survey proposed
	Prevalence of insufficient physical activity	No	
	Human papilloma virus (HPV) vaccination coverage	No	
	Percentage of women with cervical cancer screening	Yes	routine facility data
	Arthritis treatment coverage	No	
	Spectacle coverage	No	
	Dental coverage	No	
	Road traffic deaths per 100,000	Yes	Vital registration and Traffic department records.
	Harmful use (consumption) of alcohol	No	
	Current use of any tobacco product	Yes	NACADA
	Smoking cession rates	No	
	Additional indicators		
	Angina treatment coverage	No	
	Cardiovascular diseases preventive drug therapy for high risk groups	No	
	Diabetes treatment coverage	No	
	Coverage of pain relief	No	
	Asthma/COPD treatment coverage	No	
	Depression treatment coverage	No	
	Cataract surgery coverage	No	
	Coverage with rapid emergency response	No	
Equity: A measure of CCI service coverage for the poorest 40% of the population		No	

NACADA: National Authority for the Campaign against Alcohol and Drug Abuse.

Data sourced from GOK documents [25,42].

#### Financial risk protection indicators

The country has the capacity to report on the three proposed financial risk protection indicators. Table 9 illustrates the available indicators and their respective data sources. The Kenya Household and Health Expenditure and Utilization survey will be the main source of primary data for these indicators. The survey is conducted every five years and collects socio-economic data that will facilitate disaggregation of data to measure for equity. Currently the health sector does not generate the estimates for these indicators. The country relies on the out-of-pocket expenditure and the various indicators related to total health expenditure estimates to monitor health financing mechanism and policy deliberations. However, a study in the country has recently analysed the data from “*Kenya Household Health Expenditure and Utilisation survey”* to generate the estimate values of the proposed indicators [38]. Institutionalisation of these indicators within the health sector will be necessary. This will ensure that the required estimates are generated more regularly rather than on an ad hoc basis.

**Table 9:** **Available financial risk protection indicators and their data source**

**Financial risk protection coverage indicators**	**Indicator available**	**Data source**
Aggregate: a measure of the level of household impoverishment arising from out of pocket expenditures on health, equal to the ratio of the poverty gap in a world without out of pocket payments to the actual poverty gap	yes	Kenya household health expenditure and utilisation survey
Aggregate: the fraction of households incurring catastrophic out of pocket health expenditures	yes	Conducted every 5 years last survey was conducted in the year 2013
Equity: The fraction of households among the poorest 40% incurring catastrophic out-of-pocket health expenditures.	yes	

Data sourced from Ministry of Health survey report [26,28].

### Key Constraints that will affect feasibility of the framework

#### Weak health information system

The implementation of the proposed UHC monitoring framework hinges on the functionality of the country’s health information system, which will play a critical role in generating valid and reliable data that can be benchmarked and tracked to monitor UHC progress [53,54]. Currently, the country health information system experiences several challenges that impede its ability to generate the required information to meet both national and global reporting mechanisms. The health information system lacks adequate resources in terms of human resource, budget and infrastructure as well as data collection and reporting tools to conduct its functions [45]. A recent technical report suggests that the country’s health information system is not adequately responsive to meet the evolving needs for data reporting [30]. The lack of an adequate health information system has hindered the capacity of the Ministry of health to adequately steer resource allocation in line with its policy goals and objectives [21]. Consequently, the country urgently needs to address these contextual challenges. But if the implementation of UHC is driven with the necessary technical support, it will facilitate the generation of reliable health information. This has the potential to enable policy makers to identify service coverage gaps, scale up and improve health services effectively, and inform the UHC monitoring process at country and global levels.

#### Data quality

Good quality data is critical to the success of monitoring progress towards UHC. The five key critical dimensions of quality include accuracy, completeness, timeliness, consistency and accessibility [45,55]. Based on these five dimensions, a series of studies suggest that the quality of data generated at various levels of the health information system is inadequate, with reports of inaccurate data entry at facility level, incomplete data and late reporting [30,52,56,57]. There is an urgent need to address this issue since routine facility data will play a more pivotal role in the success of monitoring progress towards UHC.

#### Fragmented health information reporting system

The available indicators are captured across several data sources and programmes within the country’s health information system. The donor investments in monitoring of the health MDGs resulted in several parallel reporting channels created to meet various donor reporting needs. However, there is limited coordination across the various reporting channels [30]. In certain instances data reporting bypasses the Ministry of Health reporting channels to meet donor requirements [56]. This creates challenges in the retrieval of data for the indicators and discrepancies in data generated. The fragmentation will affect the availability of reliable, consistent and timely generation of data for the UHC indicators.

#### Intervals of data availability

The national surveys are often a preferred data source since they provide better quality data and are more generalizable to the whole population. However, the frequency of conducting these surveys is varied and hence has an important implication on the availability of data. These surveys are conducted in the country on average every 3–5 years. Furthermore, the roll out of individual surveys and the frequency of these surveys are not synchronised [30,46]. Results of different surveys measuring different aspects of UHC indictors will be available at different times. This means the country may only have the capacity to sufficiently report on some indictors every five years or more. In the event that the global monitoring process requires more frequent reporting, i.e. shorter that the five period, the country’s capacity to report will be limited to facility data.

## Conclusions

The WHO/World Bank focus on developing a comprehensive monitoring framework for UHC within the Sustainable Development Goals has exposed not only the weaknesses of the health information systems in developing countries, but also the vulnerabilities of the health systems that underpin them. The aspiration to provide access to health services to all Kenyans in a bid to spur social and economic development has been articulated in several government policies. The Ministry of Health has undertaken several piecemeal strategies to facilitate the expansion of service delivery and affordability of health services [26,30,43]. However, these policies have not been linked to appropriate operational plans and budget allocations, resulting in weak policy implementation. Health policy priorities, budgets and implementation have been significantly affected by changes in political leadership and direction [30,58,59]. Moreover, the policies have mainly focused on health financing without adequately addressing other health systems issues that limit service delivery [21,40]. The consequence of this limited policy approach undertaken with its weak policy implementation has resulted in limited progress towards UHC. Access to health services remains limited, inequitable and expensive for the majority of the population.

The problem for Kenya—and many other low and middle income countries—is cyclical. The implementation of an effective monitoring framework to monitor progress towards UHC assumes a functioning health system that can sustain effective health information data collection and reporting. There needs to be political commitment to the concept of UHC before this can happen: data collection, particularly where it is of dubious quality, will at best point to weaknesses, but not drive their reform. Lack of strong stakeholder engagement and commitment to achieving UHC remains an important impediment to Kenya’s progress [11,21]. Countries that have had high level political commitment like Ghana, Vietnam, Rwanda and Mexico have made significant progress in increasing UHC in the last decade [35]. This has facilitated the necessary leadership, multi-sectoral cooperation and budgetary allocations to propel the UHC agenda [35,60]. There is increasing evidence to suggest that even low income countries can achieve UHC, if the appropriate policy decisions and investments are undertaken [35].

In Kenya, the agenda to achieve UHC has largely been driven by the Ministry of Health, but the Ministry does not have sufficient direct influence on the country’s budget, development or political agenda to bring about the whole of government changes required for UHC [21]. The Ministry’s policies will only be feasible if it can secure the support and prioritisation of UHC by the President and Parliament of Kenya. Prioritisation of UHC in the post-2015 Sustainable Development agenda offers a global profile for UHC reforms, and an opportunity to generate stronger stakeholder commitment and momentum for implementation of UHC.

The results of this study have clear implications: with the global MDG focus on HIV/AIDS, tuberculosis and malaria, the monitoring and reporting mechanisms for those conditions have been developed and institutionalized. In contrast, the systems for reporting on chronic conditions and injuries are ad hoc, uncoordinated, inconsistently reported and not always representative. Extending the success of MDG monitoring will require not only improvements in health information systems, but the comprehensive development of strategies to address this growing non-communicable burden of disease. This has implications not only for Kenya, but also for the international community.

The implementation of MDG programmes and the internationally driven national surveys such as the KDHS, KAIS and the Kenya Malaria survey have established an infrastructure for reporting on most of these indicators. The surveys have the potential to be adapted to include CCI indicators, financial risk protection and the specific equity measures, but financial considerations will limit the frequency of their application. There is a need to set up additional routine mechanisms for monitoring CCI indicators, extending monitoring into the private sector, and investing in the health information system to enhance the generation of good quality data. This will be critical for supporting a meaningful and informative UHC monitoring and policy decision processes.

With aggregated reporting recommended in the evolving WHO/World Bank UHC monitoring framework [34], care needs to be taken that the existing capacity to report on both MDG prevention and treatment service coverage indicators does not conceal the inadequacies in reporting systems for the new focus—CCI interventions. Using the first version of the UHC framework, that distinguishes MDG and CCI service coverage indicators, we have clearly demonstrated the specific gaps in relation to CCI indicators that need to be addressed.

These results are consistent with the findings of other studies that have been conducted on monitoring UHC: other low income countries share Kenya’s limited ability to report on the indicators for CCIs [17,19]. Yet even with the monitoring of MDGs, many countries have not been able to report on selected indicators [61]. And although this study found that several of the MDG-related indicators were available in Kenya, the reliability, comprehensiveness and timeliness of the data has been a key challenge for the health information system [45,56].

As global discussions on UHC and post 2015 development agenda are ongoing, it will be critical to make plans on how the required data can be generated by Kenya and other developing countries in similar scenarios. Post-2015 planners now need to be considering how low-income countries will be supported in terms of technical expertise, financial resources, and the extensive sensitisation and training of primary data collectors on the new framework. The WHO and World Bank, if they see the need for such a level of reporting for UHC, need to consider the implications of development assistance required for such substantial infrastructure—even in emerging middle income countries like Kenya. The global community will need to engage with individual countries to establish to what extent each can realistically report on the framework, and to identify the extent of investments required.

The implementation of the proposed UHC monitoring framework in Kenya will be beneficial for the country to assess its progress. To some extent, the global focus will drive domestic planning and investment for the indicators not currently available in Kenya. Kenya, like any country, will have to make policy decisions and trade-offs on how to approach the expansion of UHC to suit its context [1]. The monitoring and assessment of the process will be necessary to steer the country’s process [60]. The framework has clearly prioritized both health service coverage and financial protection for the whole population, but for Kenya, this should not be equated merely to establishing social health insurance as captured in some of the Ministry of Health policy documents [41,47]. The focus of UHC monitoring on health outcomes, financial protection and equity has the potential to galvanize reform for policy makers in the country. In anticipation of this global momentum, Kenya needs to urgently develop a comprehensive policy framework that can pragmatically move the country forward in its quest for UHC.

## Additional files

Additional file 1
**Summary of the potential tracer indicators for aggregate MDGs-related service coverage measures.**
Additional file 2
**Summary of the potential tracer indicators for aggregate CCIs-related service coverage measures.**
Additional file 3
**List of the 25 documents used for the study.**
Additional file 4
**Revised WHO /World Bank global framework for UHC monitoring.**

## References

[1] World Health Organization (2010). The World Health Report: Health systems financing: the path to universal coverage.

[2] World Health Organization: **Sustainable health financing, universal coverage and social health insurance, in World Health Assembly Resolution** [http://www.who.int/health_financing/documents/cov-wharesolution5833/en/]. [Accessed 4/2/2014]

[3] United Nation**: UN Resolution on Universal Health Coverage** [http://www.un.org/ga/search/view_doc.asp?symbol=A/67/L.36&referer=http://www.un.org/en/ga/info/draft/index.shtml&Lang=E] [Accessed 4/2/2014]

[4] World Health Organization: **Positioning Health in the Post-2015 Development Agenda** [http://www.who.int/topics/millennium_development_goals/post2015/WHOdiscussionpaper_October2012.pdf], [Accessed 4/2/2014]

[5] Sachs JD (2012). Achieving universal health coverage in low-income settings. Lancet.

[6] O'Donnell, O.A. and A. Wagstaff: **Catastrophic payments for health care** In *Analyzing health equity using household survey data: a guide to techniques and their implementation*: World Bank Publications; 2008: 203–212

[7] Thematic Group on Health for All of the Sustainable Development Solutions Network: *Health in the framework of sustainable development technical report for the post-2015 development agenda*. Sustainable Development Solutions Network; 2014

[8] Evans DB, Marten R, Etienne C (2012). Universal health coverage is a development issue. Lancet.

[9] Frenk J, de Ferranti D (2012). Universal health coverage: good health, good economics. Lancet.

[10] Duran A, Kutzin J, Menabde N (2014). Universal coverage challenges require health system approaches; the case of India. Health Policy.

[11] Lagomarsino G, Garabrant A, Adyas A, Muga R, Otoo N (2012). Moving towards universal health coverage: health insurance reforms in nine developing countries in Africa and Asia. Lancet.

[12] World Health Organization and World Bank: Monitoring Progress towards Universal Health Coverage at Country and Global Levels: A Framework Joint WHO / World Bank Group Discussion Paper [http://www.who.int/healthinfo/country_monitoring_evaluation/UHC_WBG_DiscussionPaper_Dec2013.pdf]

[13] World Health Organization (2013). The Role of Research for Universal Heal Coverage. Research for Universal Health Coverage: World Health Report 2013.

[14] Health Systems20/20 (2012). Measuring and Monitoring Country Progress Towards Universal Health Coverage: Concepts, Indicators, and Experiences.

[15] World Health Organization: **Measurement and monitoring of universal health coverage technical meeting technical meeting in Singapore** [http://www.who.int/healthinfo/UHC_Meeting_Singapore_Sep2013_Report.pdf?ua=]. [Accessed 23/2/2014]

[16] World Health Organization (2012). Measurement of Trends and Equity in Coverage of Health Interventions in the Context of Universal Health Coverage. Meeting Report, Rockefeller Foundation Center, Bellagio.

[17] Tine J, Hatt L, Faye S, Nakhimovsky S (2014). Universal Health Coverage Measurement in a Lower-Middle-Income Context: A Senegalese Case Study.

[18] Haas S, Hatt L, Leegwater A, El-Khoury M, Wong W (2012). Indicators for Measuring Universal Health Coverage: A Five-Country Analysis (DRAFT).

[19] Alebachew A, Hatt L, Kukla M, Nakhimovsky S (2014). Universal Health Coverage Measurement in a low-Income Context: An Ethiopian Case Study.

[20] World Bank. *Data*. [Internet] 2014 [cited 2014 February 27]; Available from: http://data.worldbank.org/country/kenya#cp_wdi.

[21] Wamai R (2009). The Kenyan health system: analysis of the situation and enduring challenges. JMAJ.

[22] World Health Organization (2013). World Health Statistics 2013, Part III Global Indicators.

[23] Ministry of Medical Services (MOMS) and Ministry of Public Health and Sanitation (MOPHS): *Kenya Health Policy 2012–2030*. Nairobi: 2012

[24] Institute for Health Metrics and Evaluation: **Global Burden of Diseases, Injuries, and Risk Factors Study.** [http://www.healthdata.org/sites/default/files/files/country_profiles/GBD/ihme_gbd_country_report_kenya.pdf. [Accessed 14/2/2014]

[25] Institute for Health Metrics and Evaluation: ***Global Burden of Disease (GBD) Cause Patterns*****.** [http://vizhub.healthdata.org/gbd-cause-patterns/] [Accessed 14/2/2014]

[26] Ministry of Health, Kenya (2013). Accelerating Attainment of Health Goals: The Kenya Health Sector Strategic and Investment Plan – KHSSP July 2012 – June 2017.

[27] National Coordinating Agency for Population and Development (NCAPD) [Kenya], Ministry of Medical Services (MOMS) [Kenya], Ministry of Public Health and Sanitation (MOPHS) [Kenya], Kenya National Bureau of Statistics (KNBS) [Kenya], ICF Macro (2011). Kenya Service Provision Assessment Survey 2010.

[28] Ministry of Health, Kenya: Household Health Expenditure and Utilisation Survey Report. Nairobi: 2009.

[29] World Health Organization: *Everybody’s business-strengthening health systems to improve health outcomes: WHO’s framework for action.* Geneva: World Health Organization; 2007.

[30] Luoma M, Doherty J, Muchiri S, Barasa T, Hofler K, Maniscalco L, Ouma C, Kirika R, Maundu J: *Kenya health system assessment 2010*. Bethesda, MD: Health Systems 20/20 project; 2010.

[31] Ministry of Medical Services, Kenya, Ministry of Public Health and Sanitation Kenya, Health Systems 20/20: *Kenya National health accounts 2009/10. Bethesda, MD:* Ministry of Medical Services Ministry of Public Health and Sanitation Health Systems 20/20 project, Abt. Associates Inc.; 2011:11–18.

[32] Republic of Kenya: *The Constitution of Kenya*. Nairobi: National Council for Law Reporting; 2010: 31.

[33] World Health Organization: **Draft comprehensive global monitoring framework and targets for the prevention and control of non-communicable disease** [http://apps.who.int/gb/ebwha/pdf_files/WHA66/A66_8-en.pdf] [Accessed 23/3/2014]

[34] World Health Organization and The World Bank (2014). Monitoring Progress Towards Universal Health Coverage at Country and Global Levels Framework, Measures and Targets.

[35] Joint Learning Network for Universal Health Coverage: ***Compare Reforms***. [http://www.jointlearningnetwork.org/programs?sl=environment-jln_programs] [Accessed 6/3/2014]

[36] Kitui J, Lewis S, Davey G (2013). Factors influencing place of delivery for women in Kenya: an analysis of the Kenya demographic and health survey, 2008/2009. BMC Pregnancy Childbirth.

[37] Echoka E, Kombe Y, Dubourg D, Makokha A, Evjen-Olsen B, Mwangi M, Byskov J, Olsen ØE, Mutisya R (2013). Existence and functionality of emergency obstetric care services at district level in Kenya: theoretical coverage versus reality. BMC Health Serv Res.

[38] Chuma J, Maina T (2012). Catastrophic health care spending and impoverishment in Kenya. BMC Health Serv Res.

[39] Kenya National Bureau of Statistics (KNBS), ICF Macro: *Kenya demographic and health survey 2008–09*. Calverton, Maryland: KNBS and ICF Macro; 2010.

[40] Chuma J, Okungu V (2011). Viewing the Kenyan health system through an equity lens: implications for universal coverage. Int J Equity Health.

[41] Kutzin J (2013). Health financing for universal coverage and health system performance: concepts and implications for policy. Bull World Health Organ.

[42] National AIDS and STI Control Programme (NASCOP): *Indicators Manual*. Nairobi: NASCOP; 2010.

[43] Ministry of Health Kenya (2007). Reversing the Trends: The Second National Health Sector Strategic Plan of Kenya - NHSSP II: Midterm Review Report.

[44] Ministry of Medical Services (MOMS): Sessional Paper No. 7 of 2012 on the policy on universal health care coverage in Kenya. Nairobi; 2012

[45] Republic of Kenya: *Report for the Assessment of the Health Information System of Kenya*. Nairobi:Division of Health Management Information System[Ministry of Health]; 2008.

[46] Ministry of Health Kenya (2008). Health Sector Indicator and Standard Operating Procedures Manual for Health Workers.

[47] Centre for Intelligence Agency (CIA): ***The World Fact Book*** [https://www.cia.gov/library/publications/the-world-factbook/geos/ke.html] [Accessed 14/2/2014]

[48] Ministry of Public Health & Sanitation (2010). National Monitoring and Evaluation Plan for Division of Leprosy, Tuberculosis and Other Lung Diseases (DLTLD).

[49] Division of Malaria Control [Ministry of Public Health and Sanitation], Kenya National Bureau of Statistics, ICF Macro (2011). 2010 Kenya Malaria Indicator Survey.

[50] National Authority For The Campaign Against Alcohol And Drug Abuse (NACADA): *Rapid situation assessment of the status of drug and substance abuse in Kenya.* Nairobi: NACADA; 2012.

[51] Ministry of Medical Services (MOMS) and Ministry of Public Health and Sanitation (MOPHS): *Health Information System Policy.* Nairobi: Ministry of Medical Services and Ministry of Public Health and Sanitation; 2009.

[52] Hahn D, Wanjala P, Marx M (2012). Where is information quality lost at clinical level? A mixed-method study on information systems and data quality in three urban Kenyan ANC clinics. Glob Health Action.

[53] Murray CJL, Lopez AD, Wibulpolprasert S (2004). Monitoring global health: time for new solutions. Br Med J.

[54] AbouZahr C, Boerma T: **Health information systems: the foundations of public health. I**n Bulletin of the World Health Organization. Switzerland: World Health Organization 2005, 578–583. PMC2626318 16184276

[55] Hodge N (2012). Quality for health information: what does it mean, why does it matter, and what can be done?. Pacific Health Dialog.

[56] Odhiambo-Otieno GW (2005). Evaluation of existing district health management information systems: a case study of the district health systems in Kenya. Int J Med Inform.

[57] Chiba Y, Oguttu MA, Nakayama T (2012). Quantitative and qualitative verification of data quality in the childbirth registers of two rural district hospitals in Western Kenya. Midwifery.

[58] Lubano K, Kariuki J, Muthami L, Mutai J, Ojakaa D, Agina O, Okoth J, Muleshe S, Bwonya J, Wasunna MK (2008). Evidence-Informed Policy Making: Health Policy and Systems Issues Setting REACH-Policy Initiative Kenya Priorities for 2008–2010.

[59] Chuma J, Musimbi J, Okungu V, Goodman C, Molyneux C (2009). Reducing user fees for primary health care in Kenya: policy on paper or policy in practice?. Int J Equity Health.

[60] Knaul FM, Gonzalez-Pier E, Gomez-Dantes O, Garcia-Junco D, Arreola-Ornelas H, Barraza-Llorens M, Sandoval R, Caballero F, Hernandez-Avila M, Juan M, Kershenobich D, Nigenda G, Ruelas E, Sepulveda J, Tapiz R, Soberon G, Chertorivski S, Frenk J (2012). The quest for universal health coverage: achieving social protection for all in Mexico. Lancet.

[61] Sanga D (2011). The challenges of monitoring and reporting on the millennium development goals in Africa by 2015 and beyond. Afr Stat J.

