# Supplementary material for: Indicators for Universal Health Coverage: can Kenya comply with the proposed post-2015 monitoring recommendations?
Source: Int J Equity Health. 2014 Dec 20;13:123. doi: 10.1186/s12939-014-0123-1 (PMC4296682; doi:10.1186/s12939-014-0123-1)
Supplement: Additional file 1 — Summary of the potential tracer indicators for aggregate MDGs-related service coverage measures. [file 12939_2014_123_MOESM1_ESM.doc]

Additional file 1: Summary of the potential tracer indicators for aggregate MDGs-related service coverage measures

| Potential tracer indicators for aggregate MDGs-related interventions.  (n= 22 indicators) |
| --- |
| Need satisfied for family planning |
| Skilled birth attendance |
| DPT3 immunisation coverage |
| Serious acute child illness coverage |
| Household ownership of ITNs |
| TB treatment coverage |
| ART coverage |
| PMTCT coverage |
| Additional coverage indicators |
| Contraceptive use |
| ANC 4+ visits |
| Institutional deliveries |
| Postnatal care (Postnatal care visit within two days of childbirth (%)) |
| Measles, BCG, polio, hepatitis B, Influenza coverage among older people. |
| Suspected pneumonia treated with antibiotic |
| Diarrhoea treated with ORS |
| Coverage of exclusive breast feeding |
| IPT during pregnancy |
| Fever treated with antimalarial |
| Households with indoor residual spraying (IRS) |
| TB case detection rate |
| Male circumcision rates |
| Condom use at higher risk sex |

Sourced from Bellagio Meeting report[12]
