# Supplementary material for: Indicators for Universal Health Coverage: can Kenya comply with the proposed post-2015 monitoring recommendations?
Source: Int J Equity Health. 2014 Dec 20;13:123. doi: 10.1186/s12939-014-0123-1 (PMC4296682; doi:10.1186/s12939-014-0123-1)
Supplement: Additional file 2 — Summary of the potential tracer indicators for aggregate CCIs-related service coverage measures. [file 12939_2014_123_MOESM2_ESM.doc]

Additional file 2: Summary of the potential tracer indicators for aggregate CCIs-related service coverage measures

| Potential tracer indicators for the aggregate CCIs-related service coverage measures.  (n=27 indicators) |
| --- |
| Percentage with hypertension diagnosed and receiving treatment |
| Probability of dying between the exact ages of 30 and 70 from any of cardiovascular disease cancer diabetes or chronic respiratory disease |
| Age-standardised prevalence of diabetes(based on HbA1c levels),hypertension, cardiovascular disease and chronic respiratory disease |
| Age-standardised mean population intake of salt per day in grams in persons aged 18+ |
| Prevalence of persons aged 18+) consuming less than five total servings of fruit and vegetable per day |
| Fraction of calories from added saturated fats and sugars |
| Hepatitis B vaccination coverage |
| Percentage of the population that is overweight and obese |
| Prevalence of insufficient physical activity |
| Harmful use( consumption) of alcohol |
| Percentage of the population that is overweight and obese |
| Current use of any tobacco product (age standardised) |
| Smoking cession rates |
| Human papilloma virus (HPV) vaccination coverage |
| Percentage of women with cervical cancer screening |
| Arthritis treatment coverage |
| Spectacle coverage |
| Dental coverage |
| Road traffic deaths per 100,000 |
| Additional indicators |
| Angina treatment coverage |
| Cardiovascular diseases preventive drug therapy for high risk groups |
| Diabetes treatment coverage |
| Coverage of pain relief |
| Asthma/COPD treatment coverage |
| Depression treatment coverage |
| Cataract surgery coverage |
| Coverage with rapid emergency response |

Source Bellagio meeting report and WHO [12, 31]
