# Supplementary material for: Indicators for Universal Health Coverage: can Kenya comply with the proposed post-2015 monitoring recommendations?
Source: Int J Equity Health. 2014 Dec 20;13:123. doi: 10.1186/s12939-014-0123-1 (PMC4296682; doi:10.1186/s12939-014-0123-1)
Supplement: Additional file 3 — List of the 25 documents used for the study. [file 12939_2014_123_MOESM3_ESM.doc]

Additional file 3: List of the 25 documents used for the study

1. Chiba Y, Oguttu MA, Nakayama T**: Quantitative and qualitative verification of data quality in the childbirth registers of two rural district hospitals in Western Kenya**. Midwifery 2012, 28(3): 329-339.

2. Chuma J, Maina T: **Catastrophic health care spending and impoverishment in Kenya.** BMC health services research 2012, **12**(1): 413.

3. Chuma J, Okungu V: **Viewing the Kenyan health system through an equity lens: Implications for universal coverage**. International Journal for Equity in Health 2011, 10:22.

4. Division of Malaria Control [Ministry of Public Health and Sanitation], Kenya National Bureau of Statistics, ICF Macro: *2010 Kenya Malaria Indicator Survey*. Nairobi: DOMC, KNBS and ICF Macr; 2011:30-64.

5. Echoka E, Kombe Y, Dubourg D, Makokha A, Evjen-Olsen B, Mwangi M, Byskov J, Olsen, ØE, Mutisya R: **Existence and functionality of emergency obstetric care services at district level in Kenya: theoretical coverage versus reality***.* BMC Health Services Research 2013, 13(113): 1-9.

6. Hahn D, Wanjala P, Marx M: **Where is information quality lost at clinical level? A mixed-method study on information systems and data quality in three urban Kenyan ANC clinics***.* Glob Health Action 2012, 6: p. 21424.

7. Republic of Kenya: *Report for the Assessment of the Health Information System of Kenya*. Nairobi:Division of Health Management Information System[Ministry of Health]; 2008.

8. Ministry of Medical Services (MOMS) and Ministry of Public Health and Sanitation (MOPHS):*Health Information System Policy.* Nairobi: Ministry of Medical Services and Ministry of Public Health and Sanitation; 2009.

9. Kenya National Bureau of Statistics (KNBS), ICF Macro: *Kenya demographic and health survey 2008-09*. Calverton, Maryland: KNBS and ICF Macro; 2010.

10. Kitui, J, Lewis S, Davey G: **Factors influencing place of delivery for women in Kenya: an analysis of the Kenya demographic and health survey, 2008/2009***.* BMC Pregnancy And Childbirth 2013, 13(40): 1-10.

11. Luoma M, Doherty J, Muchiri S, Barasa T, Hofler K, Maniscalco L, Ouma C, Kirika R, Maundu J: *Kenya health system assessment 2010*.Bethesda, MD: Health Systems 20/20 project; 2010.

12. Ministry of Health Kenya: *Health sector indicator and standard operating procedures manual for health workers*. Nairobi: Ministry of Health - Health Management Information System Department; 2008.

13. Ministry of Health Kenya*: Reversing the Trends: The Second National Health Sector Strategic Plan of Kenya - NHSSP II: Midterm Review Report.* Nairobi: Ministry of Health - Sector Planning and Monitoring Department; 2007. [

](http://www.equityhealthj.com/sfx_links?ui=1475-9276-13-27&bibl=B29)

14. Ministry of Health, Kenya: Household Health Expenditure and Utilisation Survey Report. Nairobi; 2009.

15. Ministry of Health, Kenya: Accelerating Attainment Of Health Goals: The Kenya Health Sector Strategic And Investment Plan – KHSSP July 2012 – June 2017.Nairobi; Ministry of Medical Services and Ministry of Public Health & Sanitation; 2013.

16. Ministry of Medical Services, Kenya, Ministry of Public Health and Sanitation Kenya, Health Systems 20/20: *Kenya National health accounts 2009/10. Bethesda, MD:* Ministry of Medical Services Ministry of Public Health and Sanitation Health Systems 20/20 project, Abt. Associates Inc.; 2011:11-18.

17. Ministry of Medical Services (MOMS): Sessional Paper No. 7 of 2012 on the pol icy on universal health care coverage in Kenya.Nairobi; 2012.

18. Ministry of Medical Services (MOMS) and Ministry of Public Health and Sanitation (MOPHS): *Kenya Health Policy 2012-2030*. Nairobi; 2012.

19. Ministry of Publich Health & Sanitation: National monitoring and evaluation plan for division of leprosy, tuberculosis and other lung diseases (DLTLD). Nairobi: DLTLD/MOPHS; 2010: 13-28.

20. National AIDS and STI Control Programme (NASCOP): *Indicators Manual*.Nairobi: NASCOP; 2010.

21. National Authority For The Campaign Against Alcohol And Drug Abuse (NACADA): *Rapid situation assessment of the status of drug and substance abuse in Kenya.*Nairobi: NACADA; 2012.

22. National Coordinating Agency for Population and Development (NCAPD) [Kenya], Ministry of Medical Services (MOMS) [Kenya], Ministry of Public Health and Sanitation (MOPHS) [Kenya], Kenya National Bureau of Statistics (KNBS) [Kenya], ICF Macro: *Kenya Service Provision Assessment Survey 2010*. Nairobi, Kenya: National Coordinating Agency for Population and Development, Ministry of Medical Services, Ministry of Public Health and Sanitation, Kenya National Bureau of Statistics and ICF Macro; 2011.

23. Odhiambo-Otieno GW: **Evaluation of existing district health management information systems: A case study of the district health systems in Kenya*.*** International Journal of Medical Informatics 2005, 74(9): 733-744.

24. Republic of Kenya: *The Constitution of Kenya*. Nairobi: National Council for Law Reporting; 2010: 31.

25. Wamai R: **The Kenyan health system: analysis of the situation and enduring challenges**. JMAJ 2009, **52**(2): 134-140.
