# Supplementary material for: Indicators for Universal Health Coverage: can Kenya comply with the proposed post-2015 monitoring recommendations?
Source: Int J Equity Health. 2014 Dec 20;13:123. doi: 10.1186/s12939-014-0123-1 (PMC4296682; doi:10.1186/s12939-014-0123-1)
Supplement: Additional file 4 — Revised WHO /World Bank global framework for UHC monitoring. [file 12939_2014_123_MOESM4_ESM.doc]

Additional file 4: Revised WHO /World Bank global framework for UHC monitoring

| Goal | Achieve UHC. All people obtain the good-quality essential health services that they need without enduring financial hardship. |
| --- | --- |
| Targets | By 2030, all populations, independent of household income, expenditure or wealth, place of residence or gender, have at least 80% essential health services coverage. |
|  | By 2030, everyone has 100% financial protection from out-of-pocket payments for health services. |
| Health service coverage indicators. | Prevention   1. Aggregate: coverage with a set of tracer interventions for prevention services. 2. Equity: a measure of prevention service coverage as described above, stratified by wealth quintile, place of residence and gender. |
|  | Treatment   1. Aggregate: coverage with a set of tracer interventions for treatment services. 2. Equity: a measure of treatment service coverage as described above, stratified by wealth quintile, place of residence and gender. |
| Financial protection coverage indicators. | Impoverishing expenditure   1. Aggregate: fraction of the population protected against impoverishment by out-of-pocket health expenditures, comprising two types of household: families already below the poverty line on the basis of their consumption and who incur out-of-pocket health expenditures that push them deeper into poverty; and families for which out-of-pocket spending pushes them below the poverty line. 2. Equity: fraction of households protected against impoverishment or further impoverishment by out-of-pocket health expenditures, stratified by wealth quintile, place of residence and gender. |
|  | Catastrophic expenditure   1. Aggregate: fraction of households protected from incurring catastrophic out-of-pocket health expenditure. 2. Equity: fraction of households protected from incurring catastrophic out-of-pocket health expenditure stratified by wealth quintile, place of residence and gender. |

Source WHO [32]
